# Trends and Risk Factors for the Hospitalization of Older Adults Presenting to Emergency Departments After a Bed-Related Fall: A National Database Analysis

**DOI:** 10.3390/jcm14145008

**Published:** 2025-07-15

**Authors:** Andy Tom, Sergio M. Navarro, Grant M. Spears, Adam Schluttenhofer, Michelle Junker, John Zietlow, Roderick Davis, Allyson K. Palmer, Nathan K. LeBrasseur, Fernanda Bellolio, Myung S. Park

**Affiliations:** 1 Department of Surgery, Division of Trauma, Critical Care and General Surgery, Mayo Clinic, Rochester, MN 55905, USA; tom.andy@mayo.edu (A.T.); navarro.sergio@mayo.edu (S.M.N.); schluttenhofer.adam@mayo.edu (A.S.); junker.michelle@mayo.edu (M.J.); zietlow.john@mayo.edu (J.Z.); davis.roderick@mayo.edu (R.D.); 2 Department of Surgery, University of Minnesota, Minneapolis, MN 55455, USA; 3 Department of Quantitative Health Sciences, Mayo Clinic, Rochester, MN 55905, USA; spears.grant@mayo.edu; 4 Department of Medicine, Division of Hospital Internal Medicine, Mayo Clinic, Rochester, MN 55905, USA; palmer.allyson@mayo.edu; 5 Department of Physical Medicine and Rehabilitation, Mayo Clinic, Rochester, MN 55905, USA; lebrasseur.nathan@mayo.edu; 6 Department of Emergency Medicine, Mayo Clinic, Rochester, MN 55905, USA; bellolio.fernanda@mayo.edu

**Keywords:** older adult, bed-related falls, injury pattern, emergency department, hospitalization

## Abstract

**Background/objectives**: Falls are a leading cause of traumatic injury and hospitalization for adults over the age of 65. While common, bed-related falls are relatively understudied when compared to ambulatory falls. The aim of this study is to characterize the risk factors for the hospitalization of older adults presenting to U.S. emergency departments (EDs) after a fall from bed. **Methods**: This was a cross-sectional study using publicly available data from the U.S. Consumer Product Safety Commission’s National Electronic Injury Surveillance System (NEISS) from 2014 to 2023, including all adults over the age of 65 presenting to the NEISS’s participating EDs with bed-related fall injuries. We identified fall injuries using a keyword search of the NEISS narratives and determined how the fall occurred by manually reviewing a randomized 3% sample of the narratives. We summarized demographics and injury patterns with descriptive statistics. We constructed a multivariable logistic regression model to identify risk factors for hospitalization and used Poisson regression to assess temporal trends in fall incidence and hospital admissions. **Results**: An estimated average of 320,751 bed-related fall injuries presented to EDs annually from 2014 to 2023. ED visits increased by 2.85% per year, while hospital admissions rose by 5.67% per year (*p* < 0.001). The most common injury patterns were superficial injuries (contusions, abrasions, lacerations, avulsions, and punctures) (28.6%), fractures (21.7%), and internal injuries (including concussions) (21.6%). Most of the falls occurred while transitioning into or out of bed (34.4%) or falling out of bed (56.8%). Hospitalization was required in 34.1% of cases and was associated with male sex, medication use at time of injury, and fracture injuries. **Conclusions**: Bed-related falls and associated hospitalizations are increasing among older adults. ED providers should understand risk factors for hospitalization in these common injuries such as male sex, medication use at time of injury, and high-risk injury patterns. Additionally, prevention efforts should focus on helping older adults remain safely in bed and then assisting with transitions into or out of bed.

## 1. Introduction

Falls are the leading cause of injury-related morbidity and mortality in the U.S. for people over the age of 65 [1]. Older adults [defined as adults over the age of 65 are particularly susceptible to serious injury from low-energy falls due to age-related loss of bone mass and bone microstructure, reduced muscle mass, and balance impairment from other comorbidities such as diabetes [2,3,4]. Increased fall risk is also associated with an increased risk of 30-day mortality after an emergency department (ED) visit for any reason [5]. After falling, many older adults experience lasting impairment from loss of motor capacity, poor gait performance, and depression [6]. As the U.S. population ages, it is increasingly important to understand risk factors associated with falls in this patient population.

Fall injuries are also a significant burden on the finances of individual patients and society as a whole. In 2020, healthcare expenditures for fall injuries in older adults were estimated to exceed USD 80 billion, with the majority being paid by public insurers [7]. Additionally, the median cost to individuals experiencing a fall was USD 26,143, which could be a devastating blow to an already financially vulnerable demographic [8]. There are also additional costs associated with arranging long-term care or transport to follow-up appointments, as well as taking time from work for recovery.

An estimated 3 million falls presented to U.S. EDs in 2015 [9]. Many of these falls are related to consumer products that act as tripping hazards such as stairs, rugs, bathtubs, and toilets [10,11]. While some consumer product-related falls are well characterized in older adults [10,11,12], traumatic injuries secondary to falling from a bed are less well-studied. Bed-related falls have primarily been examined in a hospital or assisted living setting [13,14,15], but it is important to understand the number of these injuries that occur nationwide in the community setting. Additionally, traumatic falls from a bed may also present with injury patterns different from ambulatory falls that could guide evaluation in the ED. Lastly, it is important to understand how these injuries occur in the community setting to develop effective risk mitigation strategies.

In this study, we examined the injury patterns and demographic characteristics of older adults who presented to U.S. emergency departments following a bed-related fall from 2014 to 2023. To understand how these injuries occur and what factors lead to hospital admission, we manually reviewed a subset of narratives included in the NEISS data that provide a short description of the events preceding each injury. Additionally, we analyzed factors associated with an increased risk of hospitalization in these patients such as injury pattern, age, medication or alcohol use, and sex [16,17,18].

## 2. Materials and Methods

This was a cross-sectional descriptive study using publicly available online data from the U.S. Consumer Product Safety Commission’s National Electronic Injury Surveillance System (NEISS). The NEISS collects data on consumer product-related injuries from 100 EDs across the United States selected as a probability sample of all 5000+ U.S. hospitals. When patients with these injuries present to the ED, they are included in the NEISS database [19]. To generate national estimates, the NEISS assigns a weight to each case based on the hospital’s annual number of ED visits and geographic location [20].

At NEISS-participating hospitals, a professional coordinator collects standardized data including patient demographics (age and sex), diagnosis, body part injured, patient disposition, location where the injury occurred, and a brief narrative describing the incident that led to the injury. Additionally, the NEISS coordinator assigns each case a consumer product code that specifies a product associated with the injury [19]. Data from the NEISS has been used previously to characterize consumer product-related injury trends [10,11,12]. We adhere to the Strengthening the Reporting of Observational Studies in Epidemiology (STROBE) guidelines for reporting observational studies [21].

This study utilized publicly available data and was determined not to require Institutional Review Board (IRB) review or approval. The data used in this research is accessible to the public without any restrictions, ensuring that no identifiable information about individuals is included.

We queried the NEISS for product codes in the “Beds, mattresses, and pillows” category and included all patients ≥65 years old from 2014 to 2023 that presented after an injury to a NEISS-participating ED. We filtered for fall injuries by searching the narrative for keywords related to falls (i.e., fall, fell, etc.) and categorized bed-related injuries as “fall” or “non-fall.” We used the NEISS sampling weights to obtain national estimates and trends of bed-related fall injuries over the study period, as well as demographic and injury-related characteristics of these patients [20].

We randomly selected a sample of 3% (n = 2112) of the bed-related fall cohort for manual review. The 3% was based on the necessary sample size for estimating event proportions, based on a population of 70,391 cases, with a 2% margin of error and 95% confidence interval [22,23]. In the bed-related fall cohort, we reviewed the narrative for each injury and sorted injuries into one of the following categories:

1. Falling out of bed—incidents where the patient fell out of bed while they were already safely in bed.
2. Transitioning—falling during a transition into or out of bed.
3. During activity out of bed—falls that occurred when the patient was out of bed performing some activity (i.e., making the bed, vacuuming around the bed and tripped on bedframe, etc.).
4. Found down/other—patient was found down by someone else and unable to report mechanism or fall was due to a unique mechanism (i.e., trying to exercise sitting on edge of bed and fell).
5. Incorrectly labeled fall—injuries that were incorrectly identified as falls (n = 59, 2.8%). These were excluded from subsequent analyses.

We also reviewed the “body part” and “diagnosis” codes designated by the NEISS and combined them into the following “injury pattern” categories using a modified Barell matrix [24]: lower trunk fractures/dislocations, upper trunk fractures/dislocations, lower extremity fractures/dislocations, upper extremity fractures/dislocations, head/face/neck fractures, internal injuries (including hematomas and concussions), pain/weakness, contusions/abrasions/lacerations/avulsions/punctures, sprains/strains, and other/unspecified.

We report descriptive statistics as median (IQR) or frequency (%) and display the data three ways for comparison—as an unweighted summary of the full NEISS bed-related falls cohort, as weighted estimates for the full U.S. population based on the full cohort and each patient’s associated NEISS sample weight, and finally as an unweighted summary of the manually reviewed 3% subsample (after removing the incorrectly included non-fall injuries). To conduct a trend analysis of falls over time, within the full bed-related falls cohort, we fitted Poisson regression models to assess changes in the number of fall-related ED visits and resulting hospitalizations over time, with year as the independent variable. We adjusted these counts by dividing by the total number of ED visits per year among geriatric patients to account for annual fluctuations in total ED visits. We assessed the goodness of fit of the Poisson regression models with McFadden’s pseudo-R-squared.

Using the subsample of manually reviewed cases, we analyzed risk factors for hospitalization after presentation to the ED with logistic regression. We analyzed demographic and injury-related characteristics in a simple regression model and considered variables with a *p*-value less than 0.10 for inclusion in a multivariable regression model. Because data was only available from 2018 to 2023 regarding the contribution of medications to the fall incidents, we constructed a secondary multivariable model including this variable on data from that time frame. All statistical analyses were completed using R (version 4.3.0; R Core Team, 2024).

## 3. Results

Table 1 includes the demographics and injury characteristics of the cohort. From 2014 to 2023, a total of 70,391 patients with bed-related falls presented to NEISS-participating EDs, with an average of 7039 bed-related falls per year. This translated to a mean national estimate of 320,751 (SD 58,218) bed-related injuries per year. Over the study period, after accounting for sampling weights, the estimated median patient age was 81 (IQR 73–88), with 36.3% men and 63.7% women. An estimated 64.2% of injuries were treated and released from the ED, 35.1% required hospitalization, observation, or subsequent transfer, 0.5% left without being seen, and 0.1% resulted in mortality. Superficial injuries (contusions/abrasions/lacerations/avulsions) were the most common injury pattern with a frequency of 28.6%, fracture and dislocations accounted for 21.7%, and internal injuries (including concussions) accounted for 21.6%.

**Figure 1:**
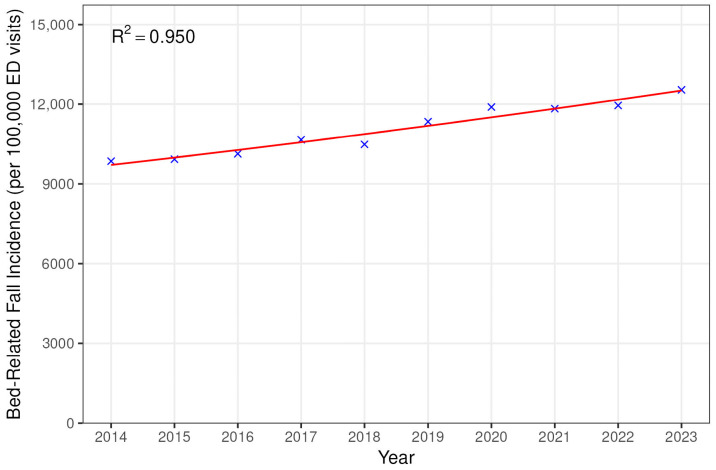
Trends in national estimated ED visits for bed-related falls.

**Figure 2:**
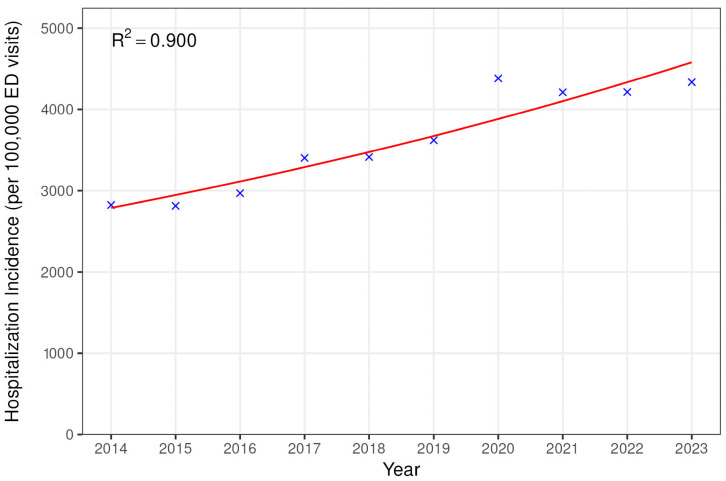
Trends in hospitalization for bed-related falls.

The number of ED visits for bed-related falls (as a fraction of total ED visits) increased by an average of 2.85% each year over the study period (95% CI 2.64–3.06%, *p* < 0.001), and hospital admissions due to falls (also as a fraction of total ED visits) increased by an average of 5.67% each year (95% CI 5.29–6.05, *p* < 0.001) (Figure 1 and Figure 2).

In the selected subsample, which was manually reviewed and categorized by mechanism of injury, a total of 56.8% of falls occurred while in bed, 34.4% occurred during a transition into or out of bed, 5.9% occurred during an activity out of the bed, and in 2.8%, the mechanism and involvement of the bed in the injury was unique or unable to be determined.

Results of the univariate logistic analyses are presented in Table 2. Men were more likely to require hospitalization and have injuries associated with medication use. Hospitalization rates were also significantly associated with injury patterns. Injury mechanism was borderline significant (*p* = 0.088), with hospitalization occurring most frequently in patients with injuries while transitioning in and out of bed. Age and alcohol use at time of injury did not meet our threshold for significance.

Based on the selection criteria (*p* < 0.10), sex, injury pattern, and injury mechanism were included in the multivariable model on all patients from 2014 to 2023 (Table 3). Men remained more likely to be admitted to the hospital compared to women (OR = 1.55, 95% CI: 1.26–1.91, *p* < 0.001). Among injury patterns, lower trunk fractures (OR = 10.04, 95% CI: 6.41–16.06 vs. patients diagnosed with pain/weakness) and lower extremity fractures (OR = 4.59, 95% CI: 2.71–7.88) were associated with the highest odds of hospitalization. Other significant injury patterns associated with increased hospitalization included other/unspecified injuries (OR = 2.52, 95% CI: 1.72–3.70), upper trunk fractures (OR = 2.23, 95% CI: 1.31–3.77), and head/neck/face fractures or dislocations (OR = 2.17, 95% CI: 1.01–4.61). Conversely, patients with superficial injuries (OR = 0.56, 95% CI: 0.39–0.80) and patients with sprains or strains (OR = 0.30, 95% CI: 0.12–0.66) were significantly less likely to be hospitalized compared to patients presenting with pain or weakness. Injury mechanism was not significantly associated with hospitalization in the multivariable model (*p* = 0.59). A secondary multivariable model (Table 4) was run on patients injured from 2018 to 2023, as data collected during this timeframe included information about whether medication/drug use contributed to each bed-related fall. In this model, falls caused by drug or medication use were associated with a higher likelihood of hospitalization (OR = 2.13, 95% CI: 1.20–3.80, *p* = 0.010).

## 4. Discussion

Our study investigated fall injuries involving a bed in older adults using a national database. Bed-related falls represent approximately 10% of the estimated 3 million falls in adults over 65 that presented to the ED in 2015 [9]. We found that bed-related fall injuries and hospitalizations have increased at a rate of 2.85% annually compared to a 3% annual increase for all consumer product-related injuries in this age group [19]. This increase mirrors a broader trend of rising fall injuries and mortality from falls [25], likely driven by increasing multimorbidity in the U.S. [26].

When examining bed-related injuries, we found that 12.8% of cases sub-sampled included ED presentations due to pain and general weakness, a category that is not part of the original NEISS coding. These cases do not fit well into a traditional injury classification scheme; however, older patients with generalized weakness are more likely to sustain an injury [27], and older patients who present to the ED with non-specific complaints have a high risk of hospitalization and 30-day mortality [28,29]. The 12.8% of patients presenting with pain/weakness in our cohort highlights the importance of a comprehensive assessment of older adults presenting to the ED after a fall and incorporating gait evaluation prior to dismissal to ensure safety [30]. Furthermore, falls in older adults represent a sentinel event and a marker for deterioration in their health [31]. Reducing fall-related injury begins with prehospital evaluations and “lift assist” calls and continues through risk assessments, screenings, and interventions that occur during and after emergency department visits [31].

A total of 36.7% of bed-related falls identified required subsequent hospitalization or transfer. Previous studies have shown that risk factors for hospitalization after a fall include diagnoses of Parkinson’s dementia and urinary tract infection, as well as low functional status and physical activity [32]. Our study found that certain injury patterns, medication use, and sex were risk factors for hospitalization. While women were more likely to present to the ED, men were more likely to require hospitalization. Men were also more likely to fall once hospitalized and are more likely to experience fall-related mortality, indicating that men may present to the hospital with more severe injuries relative to their health status than women [1,33]. Interestingly, age was not an independent risk factor for hospitalization in our cohort, indicating that other factors such as injury pattern and medication use are more important drivers of hospitalization. Other risk factors for hospitalization included fracture or dislocation of any type. These injuries were some of the most common injuries in our cohort, highlighting the need for a thorough musculoskeletal evaluation, including an evaluation for rib fractures after a bed-related fall. Injuries associated with prescription medications were also more likely to require hospitalization. The association between medications and hospitalization should prompt a medication review in older adults presenting to the ED after a fall. While internal injuries, most of which were head injuries, were not associated with an increased risk of admission in our cohort, these injuries have been associated with an increased risk of return to the ED within 90 days and should be followed up closely [34].

Polypharmacy, the use of high-risk medications like benzodiazepines or opioids [35,36], high comorbidity burden, visual and hearing impairments, orthostatic hypotension, home hazards, balance and gait abnormalities, sarcopenia, alcohol use disorder, and frailty are known risk factors for falls [37,38]. Identifying and addressing modifiable risk factors such as poor visual acuity, high-risk medication use, and gait abnormalities during ED and routine clinic visits prevent recurrent falls and promote safer mobility [39]. Measures of function collected at the index ED visit, such as the Timed Up and Go, are helpful in predicting clinical outcomes [40]. The American Geriatrics Society and the Geriatric Emergency Care Applied Research Network (GEAR) emphasize comprehensive fall risk assessments, including medication reviews and environmental evaluations, as part of their guidelines for geriatric emergency care [41].

The mechanisms we identified through our narrative analysis provide valuable insight for prevention efforts. Falling out of bed while already in bed accounted for 56.8% of injuries, while falls during a transition into or out of bed represented 34.4% of injuries. Interestingly, falls occurring while the patient was out of bed, such as tripping over the bed frame or falling while making the bed, were less frequent at 5.9% but still warrant attention as part of a comprehensive fall prevention strategy. Previous studies have demonstrated that falls are associated with decline in function in more than one third of the patients [37]. Our findings suggest that while fall mechanism was a significant risk factor for hospitalization in the univariate analysis, injury pattern, especially fractures, was the most important driver of hospitalization risk. This may be because certain fall mechanisms such as transitioning could lead to more severe injuries; thus, the mechanism and injury pattern may be dependent on one another.

While physical measures such as bed rails, compliant flooring, and low-level beds have not been shown to reduce bed-related fall injuries in hospital and long-term care settings, these interventions have not been evaluated in older adults living independently [13,14,15]. Exercise plans have been shown to reduce the risk of falling and risk of serious injury after a fall and could help reduce the risk of falling while transitioning into or out of bed [42]. Additionally, multifactorial plans that include an assessment (timed up and go test, gait speed test, Berg balance scale, etc.), a medication review, and one or more additional interventions based on individual risk factors have been shown to be effective at reducing falls, in general, but were not specifically targeted toward bed-related falls [42]. Our research suggests that interventions focused on helping older adults remain safely in their beds during rest and move in and out of their beds could have the greatest impact on reducing bed-related falls.

Our research had several limitations. The NEISS collects data from a nationally representative sample of EDs but does not include a validated injury severity score and does not account for non-emergent injuries treated at community clinics. As a result, the database likely underestimates the total incidence of bed-related fall injuries. Furthermore, the NEISS captures presentations to EDs from the community but may not capture bed-related fall injuries occurring during hospitalization. Additionally, while the narratives provide a useful source of supplementary information, they are not standardized and can vary in their content. The lack of standardized reporting in the narratives could potentially introduce non-differential misclassification bias, although the analysis of the narratives has been validated in case–control studies [43]. The coding structure of the NEISS can also lack specificity on the diagnoses [44], data quality and completeness rely on documentation available from the medical records, and the NEISS only includes drug and alcohol data starting in 2018. Lastly, the NEISS only records information during the initial presentation to the ED, precluding any long-term follow-up or analysis.

While our study provides a basic understanding of the epidemiology and risk factors for hospitalization for bed-related falls, more research is needed to find interventions effective at preventing these injuries. The current study highlights the need for targeted efforts to reduce fall-related injuries in the bed environment [42]. Additionally, ongoing research is primarily focused on preventing these injuries in hospital wards or assisted living settings [13,14,15]. Future research is needed on evaluating interventions for older adults who are at risk of falling but live independently in the community.

## 5. Conclusions

The incidence of bed-related falls and significant injuries requiring hospitalization has been increasing in the past decade. This trend will most likely continue as the U.S. population ages. Our study provides insight into the trends, mechanisms, and injury patterns of bed-related falls in the geriatric (≥65 years old) population. One third of patients who present to an ED following a bed-related fall will require subsequent hospitalization. Patients with nonspecific complaints such as pain and weakness were much more likely to require hospitalization than patients with superficial injuries, highlighting the need for comprehensive assessment in all patients presenting with falls, including those that have no evident injuries. Fracture injuries—particularly lower trunk and lower extremity fractures—alongside drug/medication use and male sex emerged as the strongest predictors of hospitalization after falls. Understanding these patterns and trends is vital to guiding patient counseling and future preventative efforts.

## Figures and Tables

**Table 1:** Demographic and injury characteristics.

		NEISS Bed-Related Falls Raw Total (n = 70,391)	Weighted U.S. Estimate (n = 3,207,432)	Manually Reviewed Sample Raw Total (n = 2053)
Age	Median (IQR)	81 (73, 88)	81 (73, 88)	81 (74-88)
Sex	Male	25,677 (36.5%)	1,163,366 (36.3%)	735 (35.8%)
	Female	44,713 (63.5%)	2,044,066 (63.7%)	1318 (64.2%)
	Intersex	1 (0.0%)	80 (0.0%)	-
Hospitalized	Yes	23,975 (34.1%)	1,054,858 (32.9%)	699 (34.0%)
	No	46,416 (65.9%)	2,152,655 (67.1%)	1354 (66.0%)
Disposition	Treated and Released	44,203 (62.8%)	2,060,395 (64.2%)	1284 (62.5%)
	Treated and Transferred	1072 (1.5%)	67,590 (2.1%)	28 (1.4%)
	Treated and Hospitalized	22,903 (32.5%)	987,268 (30.8%)	671 (32.7%)
	Held for Observation	1750 (2.5%)	72,406 (2.3%)	55 (2.7%)
	Left without Being Seen	368 (0.5%)	16,096 (0.5%)	10 (0.5%)
	Fatality	95 (0.1%)	3757 (0.1%)	5 (0.2%)
Alcohol ^1^	Yes	440 (1.1%)	19,967 (1.1%)	13 (1.1%)
	No	39,932 (98.9%)	1,805,797 (98.9%)	1177 (98.9%)
Drug/Medication Caused ^1^	Yes	1736 (4.3%)	70,302 (3.9%)	55 (4.6%)
	No	38,636 (95.7%)	1,755,462 (96.1%)	1135 (95.4%)
Injury Pattern	Lower Trunk Fracture/Dislocation	5965 (8.5%)	271,325 (8.5%)	170 (8.3%)
	Lower Extremity Fracture/Dislocation	2607 (3.7%)	119,724 (3.7%)	79 (3.8%)
	Other/Unspecified	7987 (11.3%)	358,754 (11.2%)	221 (10.8%)
	Upper Trunk Fracture/Dislocation	2282 (3.2%)	104,235 (3.2%)	79 (3.8%)
	Head/Face/Neck Fracture/Dislocation	1256 (1.8%)	53,914 (1.7%)	32 (1.6%)
	Internal Injury (incl. Concussions)	16,310 (23.2%)	692,911 (21.6%)	513 (25.0%)
	Pain/Weakness	9574 (13.6%)	416,635 (13.0%)	262 (12.8%)
	Upper Extremity Fracture/Dislocation	3081 (4.4%)	145,776 (4.5%)	106 (5.2%)
	Superficial (Contusions/Abrasions/ Lacerations/Avulsions)	18,641 (26.5%)	918,711 (28.6%)	521 (25.4%)
	Sprains/Strains	2688 (3.8%)	125,528 (3.9%)	70 (3.4%)
Injury Mechanism ^2^	In Bed (Fell Out/Rolled)	-	-	1167 (56.8%)
	Transitioning	-	-	707 (34.4%)
	Out of Bed (Walking/Activity)	-	-	122 (5.9%)
	Other/Unclear	-	-	57 (2.8%)

^1^ Data only available for fall injuries from 2018–2023. ^2^ Data only available for manually reviewed sample.

**Table 2:** Univariate analysis identifying predictors of hospitalization.

Variable	Level	N Total	N Hospitalized	% Hospitalized	OR	95% CI	*p*-Value
Age	Per 10 years	-	-	-	1.07	0.97, 1.18	0.19
Sex	Male	735	285	38.8%	1.38	1.15, 1.67	**<0.001**
	Female	1318	414	31.4%	1.00	Ref	
Alcohol ^1^	Yes	13	7	53.8%	2.00	0.66, 6.24	0.22
	No	1177	434	36.9%	1.00	Ref	
Drug/Medication Caused ^1^	Yes	55	29	52.7%	1.96	1.14, 3.39	**0.015**
	No	1135	412	36.3%	1.00	Ref	
Injury Pattern							**<0.001**
	Lower Trunk Fracture	170	133	78.2%	9.86	6.31, 15.72	
	Lower Extremity Fracture	79	48	60.8%	4.25	2.52, 7.26	
	Other/Unspecified	221	123	55.7%	2.62	1.80, 3.85	
	Upper Trunk Fracture	79	35	44.3%	2.18	1.29, 3.68	
	Head/Neck/Face Fracture/Dislocation	32	14	43.8%	2.13	0.99, 4.51	
	Internal Injury (incl. Concussions)	513	167	32.6%	1.32	0.96, 1.85	
	Pain/Weakness	262	70	26.7%	1.00	Ref	
	Upper Extremity Fracture	106	27	25.5%	0.94	0.55, 1.56	
	Superficial (Contusions/Abrasions/ Lacerations/Avulsions)	521	90	17.3%	0.57	0.40, 0.82	
	Sprains/Strains	70	7	10.0%	0.31	0.12, 0.66	
Injury Mechanism							**0.088**
	In Bed (Fell Out/Rolled)	1167	384	32.9%	1.22	0.82, 1.86	
	Transitioning	707	264	37.3%	1.48	0.98, 2.28	
	Out of Bed (Walking/Activity)	124	35	28.2%	1.00	Ref	
	Other/Unclear	57	16	28.1%	0.97	0.47, 1.93	

^1^ Data only available for fall injuries from 2018–2023. **Bold** used to denote *p*-values that are significant (<0.05).

**Table 3:** Multivariable logistic regression analysis of risk factors for hospital admission following falls using covariates with *p* < 0.10 in univariate analysis.

Predictor	Level	OR	95% CI	*p*-Value
Sex	Male	1.55	1.26, 1.91	**<0.001**
	Female	1.00	Ref	
Injury Pattern				**<0.001**
	Lower Trunk Fracture	10.04	6.41, 16.06	
	Lower Extremity Fracture	4.59	2.71, 7.88	
	Other/Unspecified	2.52	1.72, 3.70	
	Upper Trunk Fracture	2.23	1.31, 3.77	
	Head/Neck/Face Fracture/Dislocation	2.17	1.01, 4.61	
	Internal Injury (incl. Concussions)	1.30	0.94, 1.83	
	Pain/Weakness	1.00	Ref	
	Upper Extremity Fracture	0.99	0.58, 1.64	
	Superficial (Contusions/Abrasions/ Lacerations/Avulsions)	0.56	0.39, 0.80	
	Sprains/Strains	0.30	0.12, 0.66	
Injury Mechanism				0.59
	In Bed (Fell Out/Rolled)	1.27	0.81, 2.02	
	Transitioning	1.37	0.86, 2.20	
	Out of Bed (Walking/Activity)	1.00	Ref	
	Other/Unclear	1.16	0.54, 2.43	

**Bold** used to denote *p*-values that are significant (<0.05).

**Table 4:** Multivariable logistic regression analysis of risk factors for hospital admission following falls using covariates *p* < 0.10 in univariate analysis with only patients injured from 2018 to 2023 when medication data was available.

Predictor	Level	OR	95% CI	*p*-Value
Sex	Male	1.44	1.11, 1.88	**0.007**
	Female	1.00	Ref	
Drugs/Medications	Yes	2.13	1.20, 3.80	**0.010**
	No	1.00	Ref	
Injury Pattern				**<0.001**
	Lower Trunk Fracture	7.98	4.55, 14.43	
	Lower Extremity Fracture	3.35	1.74, 6.58	
	Other/Unspecified	2.05	1.29, 3.27	
	Upper Trunk Fracture	1.54	0.77, 3.06	
	Head/Neck/Face Fracture/Dislocation	1.43	0.53, 3.71	
	Internal Injury (incl. Concussions)	0.90	0.59, 1.37	
	Pain/Weakness	1.00	Ref	
	Upper Extremity Fracture	0.76	0.35, 1.55	
	Superficial (Contusions/Abrasions/ Lacerations/Avulsions)	0.57	0.36, 0.89	
	Sprains/Strains	0.15	0.02, 0.52	
Injury Mechanism				0.74
	In Bed (Fell Out/Rolled)	1.37	0.77, 2.53	
	Transitioning	1.39	0.77, 2.59	
	Out of Bed (Walking/Activity)	1.00	Ref	
	Other/Unclear	1.22	0.48, 3.03	

**Bold** used to denote *p*-values that are significant (<0.05).

## Data Availability

The data supporting the conclusions of this article are available from https://www.cpsc.gov/cgibin/NEISSQuery/home.aspx (accessed on 24 October 2024).

## Abbreviations

The following abbreviations are used in this manuscript:

NEISS	National electronic injury surveillance system
ED	Emergency department
STROBE	Strengthening the Reporting of Observational Studies in Epidemiology
IRB	Institutional Review Board
GEAR	Geriatric Emergency Care Applied Research Network
